# Iodine-124 PET quantification of organ-specific delivery and expression of NIS-encoding RNA

**DOI:** 10.1186/s13550-021-00753-2

**Published:** 2021-02-10

**Authors:** Matthias Miederer, Stefanie Pektor, Isabelle Miederer, Nicole Bausbacher, Isabell Sofia Keil, Hossam Hefesha, Heinrich Haas, Ugur Sahin, Mustafa Diken

**Affiliations:** 1 Department of Nuclear Medicine, University Medical Center of Johannes Gutenberg University, Mainz, Germany; 2 TRON - Translational Oncology at the University Medical Center, Johannes Gutenberg University Mainz gGmbH, Mainz, Germany; 3 Biopharmaceutical New Technologies (BioNTech) SE, Mainz, Germany

**Keywords:** Iodine-124, PET/MRI, Cancer vaccination, NIS, RNA vaccine, Cancer immunotherapy

## Abstract

**Background:**

RNA-based vaccination strategies tailoring immune response to specific reactions have become an important pillar for a broad range of applications. Recently, the use of lipid-based nanoparticles opened the possibility to deliver RNA to specific sites within the body, overcoming the limitation of rapid degradation in the bloodstream. Here, we have investigated whether small animal PET/MRI can be employed to image the biodistribution of RNA-encoded protein. For this purpose, a reporter RNA coding for the sodium-iodide-symporter (NIS) was in vitro transcribed in cell lines and evaluated for expression. RNA-lipoplex nanoparticles were then assembled by complexing RNA with liposomes at different charge ratios, and functional NIS protein translation was imaged and quantified in vivo and ex vivo by Iodine-124 PET upon intravenous administration in mice.

**Results:**

NIS expression was detected on the membrane of two cell lines as early as 6 h after transfection and gradually decreased over 48 h. In vivo and ex vivo PET/MRI of anionic spleen-targeting or cationic lung-targeting NIS-RNA lipoplexes revealed a visually detectable rapid increase of Iodine-124 uptake in the spleen or lung compared to control-RNA-lipoplexes, respectively, with minimal background in other organs except from thyroid, stomach and salivary gland.

**Conclusions:**

The strong organ selectivity and high target-to-background acquisition of NIS-RNA lipoplexes indicate the feasibility of small animal PET/MRI to quantify organ-specific delivery of RNA.

**Supplementary Information:**

The online version contains supplementary material available at 10.1186/s13550-021-00753-2.

## Background

Nanoparticles show promising potency as delivery vehicles for a variety of molecules, leading to fundamentally new applications and therapeutic strategies. Due to their complex chemical composition and relevant interaction with plasma proteins, the pharmacokinetic properties and delivery properties of nanoparticles are variable and remain challenging to adapt for optimal conditions [1]. Additionally, among the wide range of possible molecules where nanoparticulate delivery systems would be advantageous, large molecules such as RNA and DNA in particular add further challenges such as nucleic acid integrity and delivery into cells. Nevertheless, transporting RNA has recently become one of the most exciting approaches, opening an impressive set of new applications. Accomplishing precise RNA delivery to target tissue would serve as a versatile platform that enables easy and transient expression of any protein in principal. RNA is currently already in use to selectively activate the immune system against specific target proteins for cancer therapy [2]. Here, two main advantages of RNA-based therapy led to a promising vaccination platform against cancer: First, RNA stimulates the immune system directly by activating innate pattern recognition receptors, and second, selective proteins can be expressed transiently by antigen-presenting cells that in turn induce an antigen-specific T cell response [3, 4].

In developing nanoparticulate delivery systems for RNA, it is important to understand how the physicochemical properties of the nanoparticles influence in vivo pharmacokinetic behavior [5]. Then, the complex pharmacokinetic properties and cellular uptake of the nanoparticles and translation of protein from delivered RNA all have to be optimized. Size and surface properties determine the interaction with blood proteins and are major determinants of, for example, blood circulation time and biodistribution profile. In particular, the electric charge of the surface molecules appears to influence the pattern of organ accumulation. For RNA-lipoplex delivery systems, charge variation between RNA and the cationic lipid was shown to be the dominant physicochemical property for directing expression to either the spleen or the lung. An optimized RNA-lipid composition with a net negative charge was shown to drive systemic RNA delivery to antigen-presenting cells, particularly to spleen, and it has been translated into a delivery platform for cancer vaccination in clinical trials [2, 3, 6]. In contrast to those negatively charged RNA-lipoplexes, cationic RNA-lipoplexes showed similar size and stability but vastly different organ distribution with selective targeting of lung tissue [3, 7]. This cationic, non-affinity target mechanism has been shown to be very robust and hypothesized to also occur with negatively charged sulfated glycosaminoglycans in vascular endothelial cells in the lung [8]. At the cellular level, cationic RNA-lipoplexes targeted mainly endothelial cells (CD45^−^/CD31^+^) [7]. Within the lung itself, of the resident hematopoietic cells mainly dendritic cells and macrophages were targeted [7]. Thus, targeting of immune cells of a certain organ—in this case the lung—is well possible with these systems.

In evaluating the process of organ-specific, nanoparticle-mediated RNA delivery, in vivo reporter proteins are fundamentally important to prove feasibility and to quantify translated protein. The sodium-iodide-symporter (NIS), physiologically only expressed in thyroid tissue, gastric mucosa and salivary glands, is a widely used reporter-gene system offering the unique possibility of imaging radioactive iodine transport. NIS is a transporter protein normally located on the basolateral surface of thyroid follicular cells. After cellular uptake by thyroid cells, the iodine is oxidated and serves as a key atom in the synthesis of thyroid hormones. In the thyroid iodine uptake and metabolism are regulated by thyroid-stimulating hormone (TSH), typically leading to far higher physiological uptake than in other tissues. Given that in many organ systems, both expression of NIS and regulation of NIS activity by TSH are lacking, NIS reporter gene imaging is ideal for sensitive detection and possible quantification of protein translation from DNA or viral vectors for various purposes, including viral and cell therapies [9–12].

Incorporating the positron emitter Iodine-124 in this reporter gene system allows imaging and quantification by high-resolution Positron Emission Tomography (PET). Radioactive iodine isotopes offer attractive advantages over other reporter systems in view of the rapid renal elimination of extracellular iodine and the high tissue penetration of emitted radiation. This results in high and rapid contrast, reflecting the extent of translated NIS [13]. For small animal in vivo studies, dedicated PET systems in combination with MRI further expand the potential of quantifying the functional availability of delivered RNA-encoded protein. Iodine-124 has a 4.2-day half-life and a 25% positron branch (2.13 meV), combining suitable imaging properties with chemical identity to natural iodine.

In this study, two RNA-lipoplex systems for systemic NIS-RNA delivery were compared by small animal PET/MRI of Iodine-124 uptake. One system with net anionic charge is known to mediate translation primarily within the spleen, and the other with net positive charge is known to yield translation primarily within the lungs. Following in vivo PET/MRI as well as ex vivo biodistribution analysis we also visualized NIS-RNA expression over time and quantified Iodine-124 uptake as a measure of effective targeting and translation.

## Methods

### Mice

Female BALB/c mice (6–8 weeks, Janvier Laboratories, France) were housed under specific pathogen-free conditions in the animal care facility (Translational animal research center, Mainz) according to the guidelines of the regional animal care committee. All experiments were performed after approval in accordance with federal guidelines.

### Cell lines

The murine tumor cell lines MC38 and TC-1 were kindly provided by Ferry Ossendorp (Leiden University) and T.C. Wu (Johns Hopkins University), respectively. Cells derived from working cell banks with low passage number were used in experiments.

### RNA

Codon-optimized human NIS (NIS) and firefly luciferase (LUC) coding sequences were cloned into the pST1 DNA vector optimized for stability as well as protein translation (5`and 3´ UTRs of TEV [14] (leader translation enhancing sequence of tobacco etch virus) and FI element [15] (a combination of UTRs from AES-mtRNRl genes), respectively). ß-S-ARCA-capped RNA was generated from linearized vectors via in vitro transcription with 1-methyl-pseudouridine modification as described previously [3, 15]. An RNA construct which does not encode a protein was used as a non-coding-RNA control in some experiments.

### RNA-lipoplexes

Lipoplexes were manufactured by mixing cationic liposomes with the RNA using dedicated protocols as described previously [3, 7]. Briefly, the RNA-lipoplexes were generated by dilution of RNA in H_2_O and 1.5 M NaCl prior to addition of liposomes to obtain the ratios given below and a final NaCl concentration of 150 mM. To formulate spleen-targeting RNA-lipoplexes, liposomes consist of a 2:1 (mol:mol) mixture of the synthetic cationic lipid 1,2-di-O-octadecenyl-3-trimethylammonium propane (DOTMA) (Merck & Cie, Schaffhausen, CH) and the phospholipid 1,2-Dioleoyl-sn-glycero-3-phosphoethanolamine (DOPE) (Avanti Polar Lipids, Alabaster, AL, USA, or Corden Pharma, Liestal, CH). To make the lung-targeting RNA-lipoplexes, liposomes consisting of DOTMA and cholesterol (Avanti Polar Lipids, Alabaster, AL, USA) at a 1:1 molar ratio were used.

The charge ratio for particle formation was calculated using the molar mass of 670 Da for the positively charged DOTMA and a mean molar mass of 330 Da for each negatively charged nucleotide of the RNA. For particles targeting the lung the charge ratio was 4:1, resulting in particles with an average size of ~ 260 nm and a polydispersity index of ~ 0,23. For particles to target the spleen the charge ratio was 1.3:2, resulting in an average size of ~ 250 nm and a polydispersity index of ~ 0,25.

### Optical imaging

Mice were injected intravenously with DOTMA/Chol:RNA (4:1) or DOTMA/DOPE:RNA (1.3:2) RNA-lipoplexes including 20 µg Luc-RNA, and in vivo luminescence imaging was performed as described previously [3]. Briefly, 6 h after RNA-lipoplex injection, mice were anesthetized with isoflurane (Abbott) in a flexi glass chamber and an aqueous solution of D-Luciferin (BD Biosciences) was administered intraperitoneally. Mice were then placed in the light-tight chamber of IVIS Spectrum In Vivo Imaging System (PerkinElmer), and emitted luciferase signal was acquired for 1 min. Luciferase signal superimposed on a black-white photographic image of the mouse was presented on a rainbow color scale.

### NIS expression in vitro

2 × 10^6^ MC38 or TC-1 cells were washed twice in X-vivo medium (Lonza) and electroporated with 20 µg NIS-RNA in 4-mm cuvettes using ECM 830 electroporator (BTX Harvard Apparatus) (300 V for MC38, 260 V for TC-1, 1 pulse, 15msec for both cell lines). Cells were harvested 6, 24 and 48 h after electroporation and stained with AlexaFluor 647 labeled-human NIS antibody (1:100 dilution) for 30 min at 4 °C in the dark. Live-dead staining was performed using Fixable Viability Dye eFluor 780 (1:3000 dilution) (Thermofisher Scientific). Data were acquired with FACS Canto II flow cytometer (BD Biosciences) and analyzed by FlowJo Software ver 10.4 (Tree Star). % NIS + cells as well as mean fluorescence intensity (MFI) of NIS expression of live cells was presented. Cells electroporated without RNA served as mock control.

### PET/MRI

Groups of *n* = 3 animals were intravenously injected with RNA-lipoplexes containing 20 µg NIS RNA. Six hours later 6.64 ± 0.66 MBq Iodine-124 (DSD Pharma, Austria or PerkinElmer, Germany) was injected intravenously. Three hours after Iodine-124 injection, mice were anesthetized with 2% isoflurane and static imaging was performed over 20 min by small animal PET/MRI (Nanoscan, Mediso, Budapest, Hungary). For anatomic imaging MRI measurements (Material Map for coregistration of the PET scan; 3D Gradient Echo External Averaging (GRE-EXT), Multi Field of View (FOV); slice thickness: 0.6 mm; TE: 2 ms; TR: 15 ms; flip angle: 25 deg) were performed afterward. Additionally, one animal per group was imaged dynamically for one hour. PET data were reconstructed with Teratomo 3D (4 iterations, 6 subsets, voxel size 0.4 mm), co-registered to the MR and corrected for decay. Quantitative analysis was done with the PMOD software package (version 4.1). Volumes of interests were based on anatomical MRI, and counts/ml were derived for static and dynamic PET data sets. Mean activity concentrations were calculated based on a cross-calibration factor.

All mice were killed at 3 h post Iodine-124 injection (9 h after NIS-RNA lipoplex injection) to measure ex vivo organ activity. Organs were collected and measured in a cross-calibrated gamma counter (Wizard2 2470, PerkinElmer).

## Results

### Expression kinetics of NIS-RNA in vitro

**Fig. 1:**
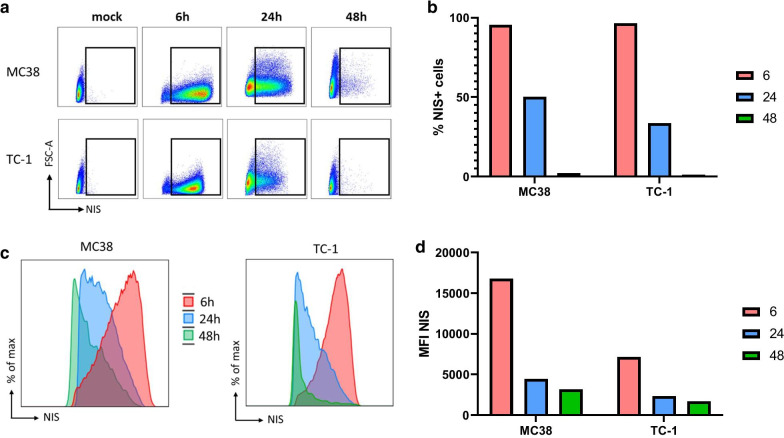
Expression kinetics of NIS-RNA in vitro. **a–d** Flow cytometric analysis of NIS expression in MC38 and TC-1 cell lines at 6, 24 and 24 h after electroporation with NIS-RNA. All data gated on live cells. **a** Representative dotplots of each cell line at indicated timepoints. **b** Percentage of NIS + cells at indicated timepoints. **c, d** The magnitude of NIS expression at the indicated timepoints is shown as representative histograms (**c**) and cumulative MFI (**d**)

In order to assess whether NIS can serve as a reporter for imaging the biodistribution of RNA-encoded protein upon intravenous administration of RNA-lipoplexes, we first cloned the human NIS gene into our DNA vector backbone and generated NIS-RNA by in vitro transcription. Transfection of this RNA into standard tumor cell lines MC38 and TC-1 via electroporation showed successful expression of NIS on the cell surface (Fig. 1a). Interestingly, due to the transient nature of RNA, the percentage of NIS-expressing cells decreased over time for both cell lines. Among the tested time points, the magnitude of NIS-expressing cells peaked at 6 h after transfection, almost halved at 24 h after electroporation, and very few NIS-positive cells were detectable after 24 h (Fig. 1a, b). In line with that, expression levels of NIS on the cell surface followed a similar pattern for both cell lines, peaking at 6 h after transfection (Fig. 1c, d). Cell viability after electroporation was > 85% (data not shown). These results show that NIS-RNA can be translated and transferred to cell membranes without causing significant toxicity in the systems tested. Moreover, 6 h after RNA transfection was selected for sampling in in vivo experiments as it seemed to be the optimum time point among those tested for NIS protein detection.

### PET/MRI after NIS-RNA lipoplex injection

**Fig. 2:**
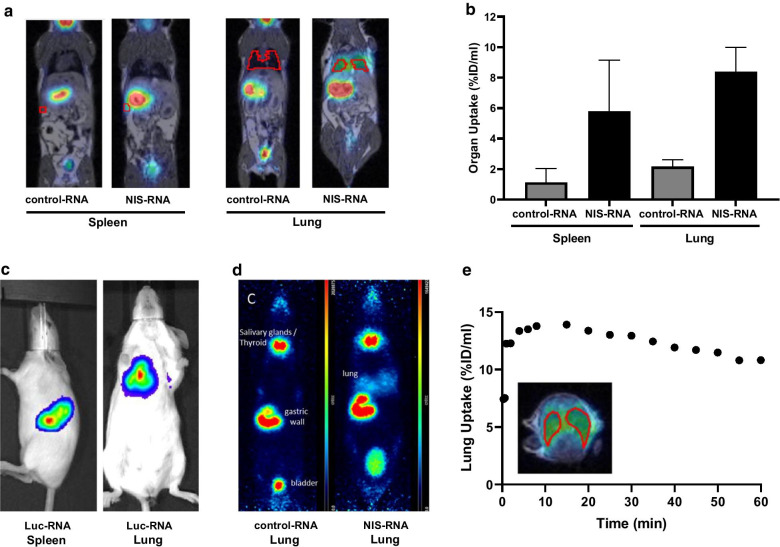
PET/MRI of Iodine-124 distribution in vivo. **a, b, d, e** Mice were inoculated i.v. with RNA-lipoplexes carrying NIS- or control-RNA, with spleen- or lung-targeting properties. Six hours later, Iodine-124 was administered and uptake was imaged and quantified 3 h after injection. **c** Alternatively, mice were injected i.v. with RNA-lipoplexes carrying Luc-RNA and in vivo luminescence imaging of the light emitted by luciferase activity was performed 6 h after. **a** Coronal slices of PET/MRI fusion and volumes of interests (red) for spleen and lung are shown in representative animals. From left to right: targeting of spleen with non-coding RNA, targeting of spleen with NIS RNA, targeting of lung with non-coding RNA, targeting of lung with NIS-RNA. **b** Calculated organ uptake from the volumes of interests. Data are shown as mean + SD of *n* = 3 mice. **c** Representative in vivo bioluminescence images of Luc-RNA lipoplexes after targeting the spleen and lung. **d** Maximum intensity projections of PET images after application of lung-targeting NIS-RNA lipoplexes (right) in comparison with non-coding control (left) **e** Time activity curve of Iodine-124 uptake in the lung over 60 min immediately after Iodine-124 injection (6 h after administration of NIS-RNA lipoplexes targeting the lung)

We inoculated mice intravenously with NIS- or non-coding (control) RNA-delivering RNA-lipoplexes with spleen- or lung-targeting properties and imaged them with PET/MR. For this purpose, Iodine-124 was administered 6 h after RNA-lipoplexes as selected above and Iodine-124 uptake was imaged and quantified 3 h after Iodine-124 injection. PET/MRI of anionic NIS-RNA lipoplexes with spleen-targeting properties showed a visually detectable increase of Iodine-124 uptake in the spleen compared to control-RNA lipoplexes (Fig. 2a, left). Due to the high physiological NIS expression in the adjacent gastric wall, this increase was only visually clear with anatomical correlation by MRI. On PET imaging, spleen uptake appeared as an irregularity of the gastric wall which is not detected in control animals (Additional file 1: Fig. 1). In contrast, lung uptake of NIS-RNA transported by cationic RNA-lipoplexes was depicted more clearly due to larger organ size and no adjacent physiological NIS uptake (Fig. 2a, right). The quantified radioactivity from imaging matched well with the extent of uptake as measured in organs ex vivo, showing enhanced uptake of NIS-RNA and expression of functional NIS-protein in lung or spleen compared to the control RNA (Fig. 2a, b). This is in line with the observation made with spleen- and lung-targeting RNA-lipoplexes carrying firefly luciferase RNA (Luc-RNA), assessed via in vivo luminescence imaging (Fig. 2c).

Interestingly, after i.v. injection of Iodine-124, the uptake in lung was rapid and remained high over the first hour of dynamic acquisition (Fig. 2d, e). This reflects the known rapid iodine uptake by NIS-expressing tissues and is in line with the in vitro measurements showing highest uptake at 6 h after NIS transfection.

### Biodistribution of Iodine-124 after NIS-RNA lipoplex Injection

**Fig. 3:**
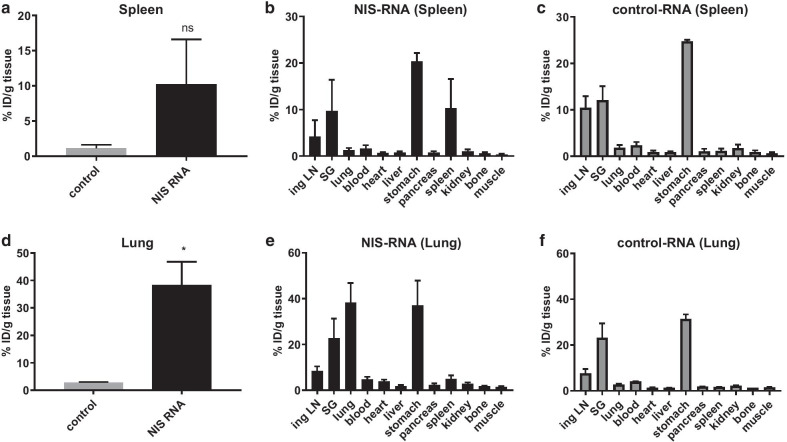
Ex vivo quantification of organ iodine uptake. **a–f** Iodine-124 uptake in organs was measured 9 h after RNA-lipoplex injection (3 h after Iodine-124 injection). Organs were weighed and % ID calculated from counts on a cross-calibrated gamma counter. **a** Radioactive uptake as a result of anionic RNA-lipoplexes targeting the spleen. **b, c** Biodistribution of radioactivity as a result of anionic (spleen-targeting) RNA-lipoplexes carrying NIS-RNA (**b**) or non-coding RNA (**c**). **d** Radioactive uptake as a result of cationic RNA-lipoplexes targeting the lung. **e, f** Biodistribution of radioactivity as a result of cationic (lung-targeting) RNA-lipoplexes carrying NIS-RNA (**e**) or non-coding RNA (**f**). Data are shown as mean + SD of *n* = 3. *SG* salivary gland, *ing LN* inguinal lymph node

After PET/MRI, mice were euthanized and organs of interest were excised, weighed and the Iodine-124 content was quantified by gamma-counting. A markedly increased accumulation of iodine occurred in spleens of mice that received spleen-targeting RNA-Lipoplexes compared to control RNA (Fig. 3a). The biodistribution of iodine uptake shows that anionic targeting to the spleen leads to strong increase of radioactivity in this organ due to NIS expression, which is comparable to physiologic uptake in the salivary glands (Fig. 3b, c). Besides accumulation in the stomach and salivary glands, both are which are known to highly express NIS per se, and all other organs only accumulated background levels of Iodine-124 (Fig. 3b, c).

The positively charged RNA-lipoplexes designed to target lung tissue induced a significantly increased accumulation of Iodine-124 in lungs of treated mice compared to control mice (Fig. 3d). Moreover, the biodistribution of iodine uptake shows that cationic targeting to the lung leads to a strong increase of radioactivity due to NIS expression (Fig. 3e, f). All other organs except stomach and salivary gland only showed background levels, although a small increase of Iodine-124 accumulation was observed in the spleen (Fig. 3e, f). These results indicate that PET/MRI can be used to track RNA-encoded protein expression with NIS as a reporter.

## Discussion

NIS as a gene therapy tool has been proposed as a potential treatment option in cancers such as hepatocellular cancer, due to the availability of a beta radiation-emitting iodine isotope (Iodine-131) which can be used as radioiodide therapy. Moreover, nanoparticulate delivery by polyplexes consisting of polymers (linear polyethylene imine-polyethylene glycol) and NIS-cDNA-targeting EGFR alone or together with cMET has been investigated [16, 17]. By using the positron emitter Iodine-124 and PET imaging 48 h after i.v. administration of gene therapy, therapy-specific Iodine-124 uptake in liver tumors modified by NIS administration was demonstrated [14]. Quantitatively, Iodine-124 uptake in tumor tissue reached 4.9% ID at 1 h post injection. Due to the conveniently long, several day half-life of radioactive iodine isotopes, NIS reporter gene imaging has also been used for monitoring macrophage migration toward inflamed tissue [12]. However, iodine efflux from cells with induced NIS expression has been shown contribute to signal decline after a few hours [18]. A second caveat to this method is that tracking uptake and expression in the spleen is significantly hampered due to physiologic NIS expression in the adjacent gastric wall.

Systemic RNA delivery with RNA-lipoplex nanoparticles provides a versatile platform that can be utilized for a number of different prophylactic and therapeutic interventions. For example, it has already been used for cancer vaccination [2, 3] as well as augmenting immune responses upon adoptive transfer of CAR-T cells [19]. To further understand the specificity of targeting and subsequent protein expression, in this study we employed a well-known model of NIS reporter gene expression. We achieved translation of NIS-encoding RNA into cell surface protein in vitro and confirmed that high organ specificity can be achieved by changing the charge ratio of the RNA-lipoplex, as detected via PET/MRI using NIS-encoding RNA. In particular, the accumulation of cationic particles in lung tissue correlated with a remarkably high accumulation of Iodine-124 in this organ. PET imaging at 3 h after Iodine-124 injection specifically showed both physiological and induced NIS expression with high contrast over background, highlighting the advantage of rapid renal excretion of iodine from the blood stream. Furthermore, it was possible to image a small organ like the spleen. Nevertheless, anatomical co-registration by PET/MRI seems necessary to discriminate spleen uptake from adjacent physiological stomach uptake. The slight increase in spleen uptake that occurred when targeting the lung with cationic RNA-lipoplexes points to minimal cross-reactivity between lung and spleen uptake, which is not detected vice versa. One strength of PET over optical imaging is the exclusion of relevant off-target protein expression, since tissue penetration of radiation is almost complete in mice. Furthermore, although optical imaging such as bioluminescence also detected RNA-encoded firefly luciferase expression, by virtue of the ability to measure discrete radioactive units, PET offers a “truer” quantification of the signals obtained from organs.

## Conclusions

We show highly specific targeting, delivery and expression of RNA to spleen and lung by anionic and cationic RNA-lipoplex nanoparticles, respectively, through the use of the NIS reporter gene system and Iodine-124 uptake as imaged by PET/MRI. Combining NIS reporter gene imaging with in vivo small animal PET/MRI thus represents a powerful tool to monitor the distribution and extent of expression of RNA targeted specifically to any tissue over time.

## Supplementary Information

**Additional file 1**.

## Data Availability

The datasets supporting the conclusions of this article are included within the article. Please contact author for further data requests.

## Abbreviations

NIS: Sodium-iodide-symporter
TSH: Thyroid-stimulating hormone
PET: Positron Emission Tomography
LUC: Firefly luciferase
DOTMA: 1,2-Di-*O*-octadecenyl-3-trimethylammonium propane
DOPE: 1,2-Dioleoyl-sn-glycero-3-phosphoethanolamine
Chol: Cholesterol
MFI: Mean fluorescence intensity
