# Supplementary material for: Iodine-124 PET quantification of organ-specific delivery and expression of NIS-encoding RNA
Source: EJNMMI Res. 2021 Feb 10;11:14. doi: 10.1186/s13550-021-00753-2 (PMC7876195; doi:10.1186/s13550-021-00753-2)
Supplement: Supplementary file 1 — Additional file 1. [file 13550_2021_753_MOESM1_ESM.pptx]

## Slide 1
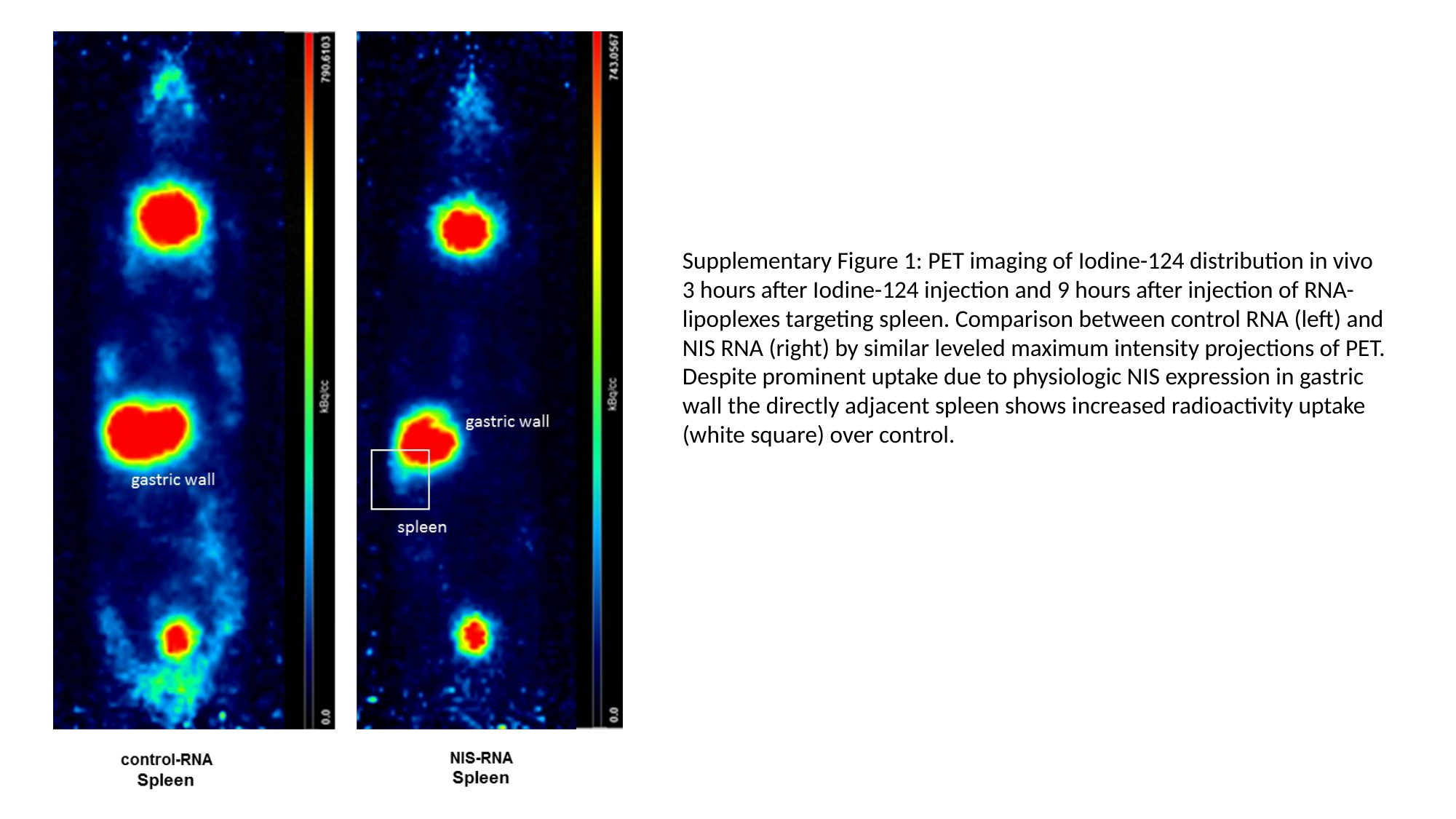

Supplementary Figure 1: PET imaging of Iodine-124 distribution in vivo 3 hours after Iodine-124 injection and 9 hours after injection of RNA-lipoplexes targeting spleen. Comparison between control RNA (left) and NIS RNA (right) by similar leveled maximum intensity projections of PET. Despite prominent uptake due to physiologic NIS expression in gastric wall the directly adjacent spleen shows increased radioactivity uptake (white square) over control.
